# The Interferon-Inducible Human PLSCR1 Protein Is a Restriction Factor of Human Cytomegalovirus

**DOI:** 10.1128/spectrum.01342-21

**Published:** 2022-02-09

**Authors:** Hidetaka Sadanari, Masaya Takemoto, Tomoki Ishida, Hikaru Otagiri, Tohru Daikoku, Tsugiya Murayama, Shuichi Kusano

**Affiliations:** a Department of Pharmaceutical Life Sciences, Faculty of Pharmaceutical Sciences, Hokuriku University, Ishikawa, Japan; b Research Center for Pharmaceutical Education, Faculty of Pharmaceutical Sciences, Hokuriku University, Ishikawa, Japan; c Division of Biological Information Technology, Joint Research Center for Human Retrovirus Infection, Kagoshima University, Kagoshima, Japan; Kumamoto University

**Keywords:** human cytomegalovirus, phospholipid scramblase 1, interferon-stimulated genes, restriction factors, genome editing, protein-protein interactions

## Abstract

Human phospholipid scramblase 1 (PLSCR1) is strongly expressed in response to interferon (IFN) treatment and viral infection, and it has been suggested to play an important role in IFN-dependent antiviral responses. In this study, we showed that the levels of human cytomegalovirus (HCMV) plaque formation in OUMS-36T-3 (36T-3) cells with high basal expression of PLSCR1 were significantly lower than those in human embryonic lung (HEL) cells with low basal expression of PLSCR1. In addition, the levels of HCMV plaque formation and replication in PLSCR1-knockout (KO) 36T-3 cells were significantly higher than those in parental 36T-3 cells and were comparable to those in HEL cells. Furthermore, compared to that in PLSCR1-KO cells, the expression of HCMV major immediate early (MIE) proteins was repressed and/or delayed in parental 36T-3 cells after HCMV infection. We also showed that PLSCR1 expression decreased the levels of the cAMP-responsive element (CRE)-binding protein (CREB)•HCMV immediate early protein 2 (IE2) and CREB-binding protein (CBP)•IE2 complexes, which have been suggested to play important roles in the IE2-mediated transactivation of the viral early promoter through interactions with CREB, CBP, and IE2. Interestingly, PLSCR1 expression repressed CRE- and HCMV MIE promoter-regulated reporter gene activities. These observations reveal, for the first time, that PLSCR1 negatively regulates HCMV replication by repressing the transcription from viral MIE and early promoters, and that PLSCR1 expression may contribute to the IFN-mediated suppression of HCMV infection.

**IMPORTANCE** Because several IFN-stimulated genes (ISGs) have been reported to suppress HCMV replication, HCMV replication is thought to be regulated by an IFN-mediated host defense mechanism, but the mechanism remains unclear. PLSCR1 expression is induced in response to viral infection and IFN treatment, and PLSCR1 has been reported to play an important role in IFN-dependent antiviral responses. Here, we demonstrate that HCMV plaque formation and major immediate early (MIE) gene expression are significantly increased in PLSCR1-KO human fibroblast cells. PLSCR1 reduces levels of the CREB•IE2 and CBP•IE2 complexes, which have been suggested to play important roles in HCMV replication through its interactions with CREB, CBP, and IE2. In addition, PLSCR1 expression represses transcription from the HCMV MIE promoter. Our results indicate that PLSCR1 plays important roles in the suppression of HCMV replication in the IFN-mediated host defense system.

## INTRODUCTION

Human cytomegalovirus (HCMV), also known as human herpesvirus 5, belongs to the *Betaherpesvirinae* subfamily. HCMV infection is generally asymptomatic in healthy individuals; however, HCMV causes severe diseases with high morbidity and mortality in both primary and recurrent infections in newborn infants and immunocompromised patients (1–3). Interferons (IFNs) are a family of related cytokines that play important roles in antiviral, antitumor, and immunomodulatory activities (4). HCMV is known to evade host immune mechanisms by suppressing IFN signaling through a variety of mechanisms (5). However, several studies have indicated that HCMV replication is inhibited by pretreatment with type I IFN (α, β) and/or type II IFN (γ) (6, 7) and is enhanced in IFN-deficient cells (8). In addition, recent studies have reported that several IFN-stimulated genes (ISGs) are involved in the IFN-mediated repression of HCMV (9–12). These observations strongly suggest that HCMV replication must be controlled by the IFN-mediated host defense system; however, the precise mechanisms are poorly understood.

Human phospholipid scramblase 1 (PLSCR1) was identified as an enzyme involved in the calcium-dependent, nonspecific, rapid redistribution of phospholipids (13). However, several studies have suggested that PLSCR1 is not involved in phospholipid redistribution when a disruption of plasma membrane asymmetry is required following cell activation or apoptosis (14, 15). Furthermore, recent studies have suggested that PLSCR1 inhibits tumorigenesis, promotes apoptosis, and facilitates the differentiation of myeloid cells through its interactions with several signaling molecules, including the epidermal growth factor receptor, src, shc, c-Abl, and onzin (16, 17). Human PLSCR1 expression is strongly induced in response to viral infection and either type I or type II IFN treatment (4, 18–20). In addition, PLSCR1 has been reported to play an important role in IFN-dependent antiviral responses (18). However, the precise antiviral mechanisms of PLSCR1 remain unclear. Recently, we and other groups reported that PLSCR1 directly interacts with and affects the function of several viral proteins (19–23), which may play a key role in PLSCR1-mediated antiviral activity.

In this study, we show that the levels of HCMV plaque formation and replication in PLSCR1-knockout (KO) human fibroblast cells are significantly increased compared to those in parental cells. In addition, compared to that in PLSCR1-KO cells, the expression of major immediate early (MIE) proteins is repressed and/or delayed in parental 36T-3 cells after HCMV infection. Interestingly, PLSCR1 specifically interacts with HCMV immediate early proteins 1 (IE1) and 2 (IE2) *in vivo*. PLSCR1 reduces the levels of the cAMP-responsive element (CRE)-binding protein (CREB)•IE2 and CREB-binding protein (CBP)•IE2 complexes, which have been suggested to play important roles in HCMV replication through its interactions with CREB, CBP, and IE2. In addition, PLSCR1 expression represses transcription from CRE- and HCMV MIE promoter-regulated reporter plasmids. These results indicate that PLSCR1 plays an important role in the suppression of HCMV replication in the IFN-mediated host defense system by repressing transcription from viral MIE and early promoters.

## RESULTS

### The expression pattern of PLSCR1 induced by HCMV infection differs in human embryonic lung and OUMS-36T-3 cells.

**FIG 1:**
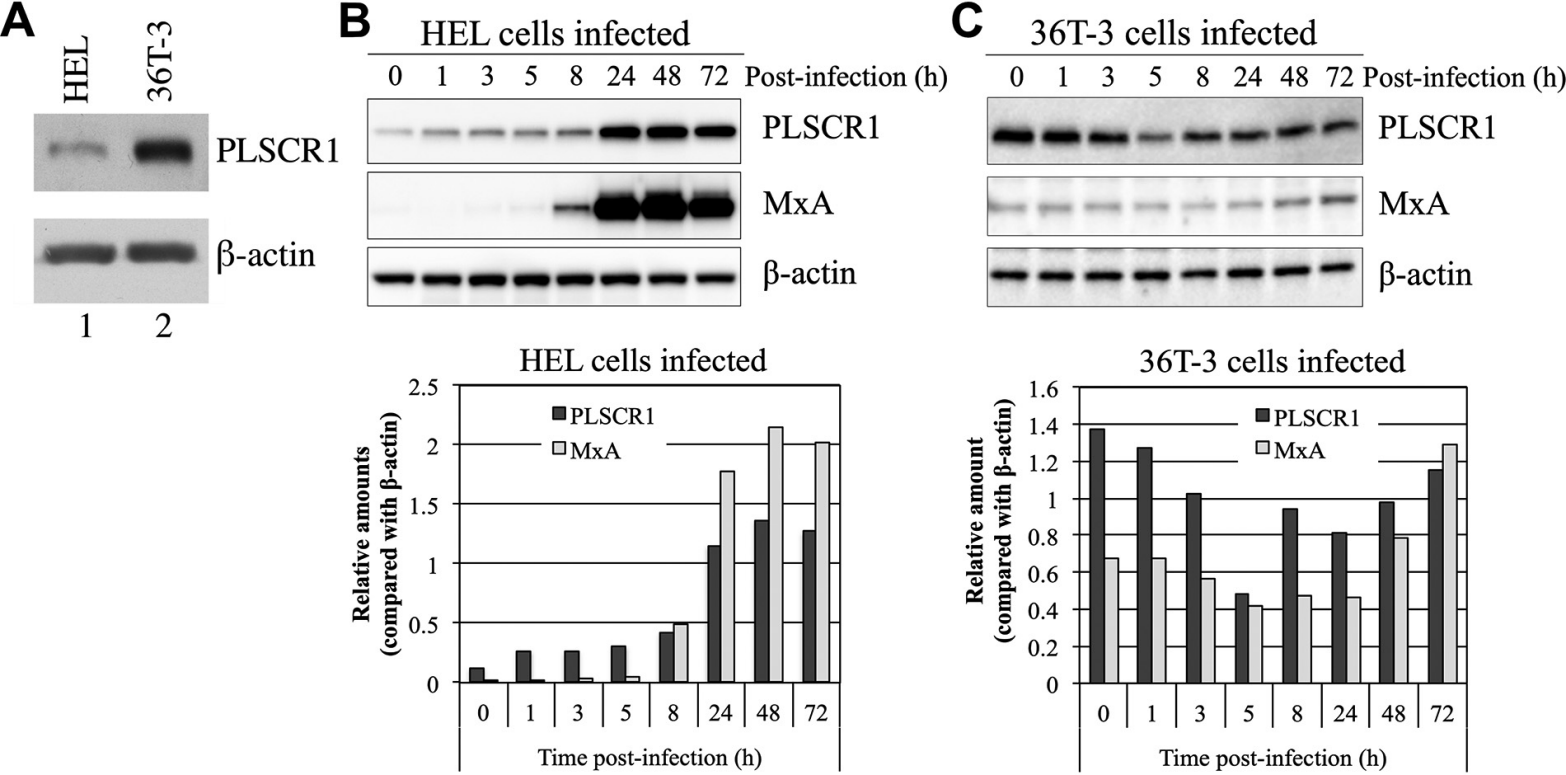
The expression level and pattern of PLSCR1 induced by HCMV infection differs in HEL and OUMS-36T-3 (36T-3) cells. (A) Total cell lysates were prepared using RIPA buffer, and a total of 5 μg of total cell lysates were subjected to SDS-PAGE. Immunoblotting (IB) was performed using an anti-PLSCR1 antibody to detect endogenous PLSCR1 or an anti-actin antibody for endogenous β-actin. (B) HEL cells were infected with the HCMV AD169 strain at a multiplicity of infection (MOI) of 3 PFU per cell, and cell lysates were prepared at the indicated time points (hours postinfection). Equivalent amounts of lysates were subjected to SDS-PAGE. IB was performed using an anti-PLSCR1 antibody to detect endogenous PLSCR1 or an anti-MxA antibody to detect endogenous MxA. Then, endogenous β-actin, as a loading control, was detected by reprobing the same membrane with an anti-actin antibody (upper panel). Band intensity was quantified using NIH ImageJ software, and levels were normalized to those of the internal β-actin control (lower panel). (C) 36T-3 cells were infected with HCMV AD169, and cell lysates preparation, IB, and quantification of band intensity were performed as described in panel B.

Human PLSCR1 expression has been shown to be induced in response to viral infection (18, 20). First, the basal expression of PLSCR1 was determined in the HCMV replication-permissive human fibroblast cell lines human embryonic lung (HEL) and OUMS-36T-3 (36T-3). The basal expression of PLSCR1 was significantly higher in 36T-3 cells than in HEL cells (Fig. 1A). Next, we determined the HCMV infection-mediated induction of PLSCR1 expression in HEL and 36T-3 cells. In HEL cells, the basal expression of PLSCR1 was significantly lower in the absence of HCMV infection. However, PLSCR1 expression was drastically induced from 24 h postinfection (p.i.) with HCMV, and this induction pattern was similar to that of another human ISG, MxA (Fig. 1B). Interestingly, the basal expression of PLSCR1 in 36T-3 cells was significantly higher than that in HEL cells in the absence of HCMV infection. However, the expression of PLSCR1 slightly decreased up to 5 h p.i. with HCMV and then gradually recovered, and this induction pattern was also similar to that of MxA (Fig. 1C). These observations indicated that the basal expression of ISGs, especially PLSCR1, in 36T-3 cells was relatively high and that their expression was affected by HCMV infection.

### Generation of PLSCR1-knockout 36T-3 cells.

**FIG 2:**
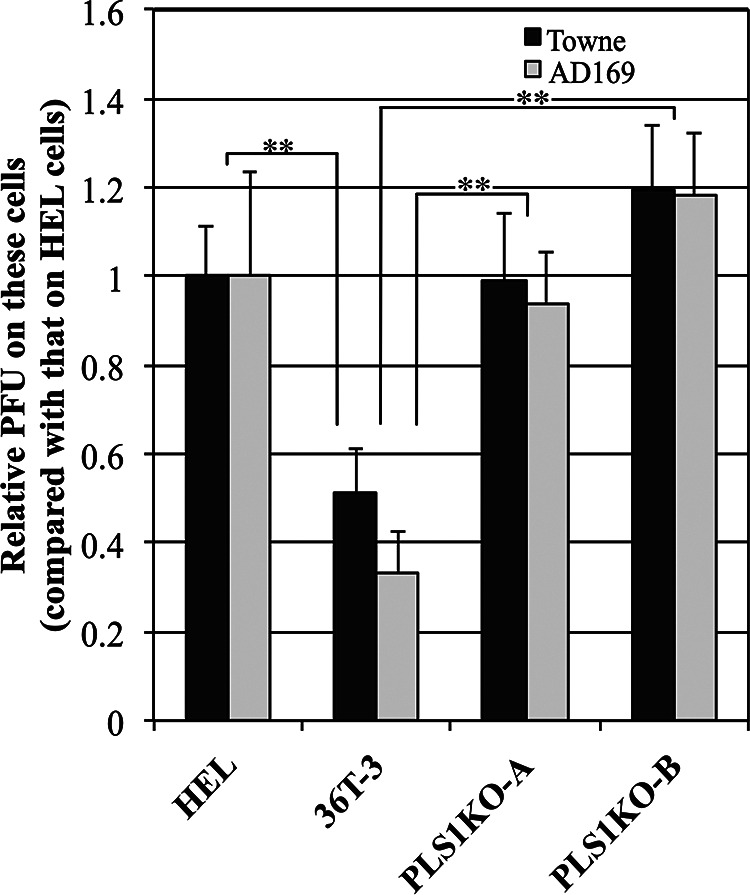
HCMV plaque formation is increased in PLSCR1-KO cells compared to parental cells. HEL, 36T-3, PLS1KO-A, and PLS1KO-B cells were infected with a 10-fold series of diluted HCMV and incubated for 6 to 10 days. The cell monolayer was fixed and then stained with methylene blue. Plaques were counted microscopically under low power, and the PFU were measured. The Data represent the average relative values from four experiments with duplicate cultures per experiment, and the error bars indicate the standard deviations. **, *P* < 0.01 by Student's *t* test.

**FIG 3:**
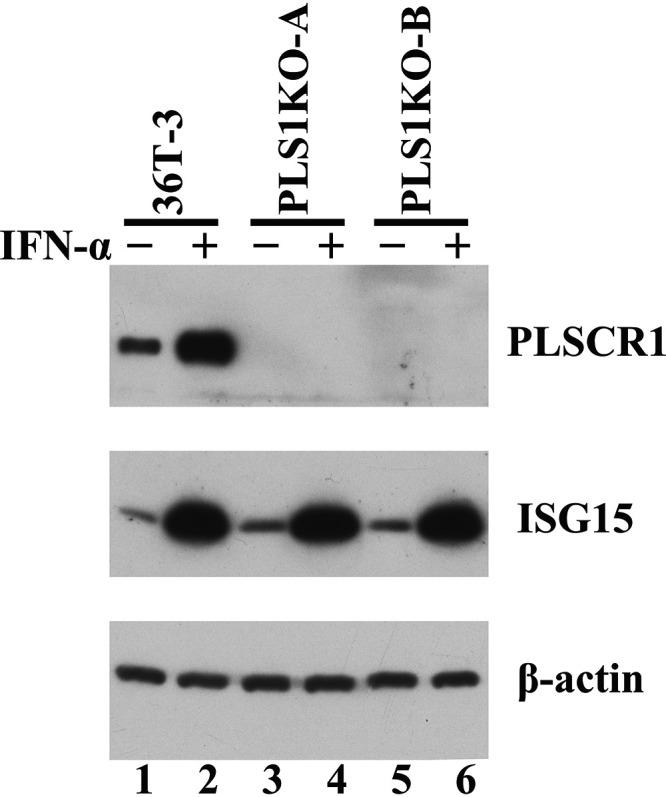
Generation of PLSCR1-KO 36T-3 cells. 36T-3 and CRISPR/Cas9-generated PLSCR1-KO 36T-3 (PLS1KO-A and PLS1KO-B) cells were treated with or without 3,000 units/mL IFN-α-2b for 16 h. Total cell lysates were prepared using RIPA buffer (Sigma-Aldrich), and a total of 4 μg of total cell lysates were subjected to SDS-PAGE. IB was performed using an anti-PLSCR1 antibody to detect endogenous PLSCR1, an anti-ISG15 antibody to detect endogenous ISG15, or an anti-actin antibody to detect endogenous β-actin.

Our observations revealed that the levels of plaque formation in 36T-3 cells due to HCMV infection were lower than those in HEL cells (shown in Fig. 2). To determine whether the relatively high levels of basal PLSCR1 expression affect HCMV growth in 36T-3 cells, we generated PLSCR1-knockout (KO) 36T-3 cells by using the CRISPR/Cas9 genome editing system. In parental 36T-3 cells, the basal expression of PLSCR1 was lower in the absence of IFN treatment, and IFN-α treatment significantly induced PLSCR1 expression (Fig. 3, top panel, lanes 1 and 2). However, no PLSCR1 expression was detected in either PLSCR1-KO cell line (PLS1KO-A and PLS1KO-B) in the absence or presence of IFN-α (Fig. 3, top panel, lanes 3 to 6). In addition, the levels of IFN-α-induced ISG15 expression were identical in the parental and PLSCR1-KO cell lines (Fig. 3, middle panel, lanes 1 to 6), consistent with our previous observation using parental and PLSCR1-KO HEK-293 cell lines (19). These observations confirmed that two 36T-3-derived PLSCR1-KO cell lines were established and that PLSCR1 is not involved in the IFN-α-mediated induction of ISG expression in these cell lines.

### HCMV plaque formation is increased in PLSCR1-KO cells compared to parental cells.

To investigate whether PLSCR1 affects the growth of HCMV in human fibroblast cells, HEL, 36T-3, PLS1KO-A, and PLS1KO-B cells were infected with two HCMV strains (Towne and AD169) diluted 10-fold in series. After 6 to 8 and 10 to 12 days of infection in HEL and 36T-3 cells, respectively, the levels of plaque formation by both HCMV strains in 36T-3 cells were significantly lower than those in HEL cells (Fig. 2). However, the levels of plaque formation in PLS1KO-A and PLS1KO-B cells were increased approximately 2-fold compared to those in parental cells, which are comparable to HEL cells (Fig. 2). These observations indicated that endogenous PLSCR1 expression reduces HCMV growth in 36T-3 cells.

### HCMV replicates to higher titers in PLSCR1-KO cells than in parental cells.

**FIG 4:**
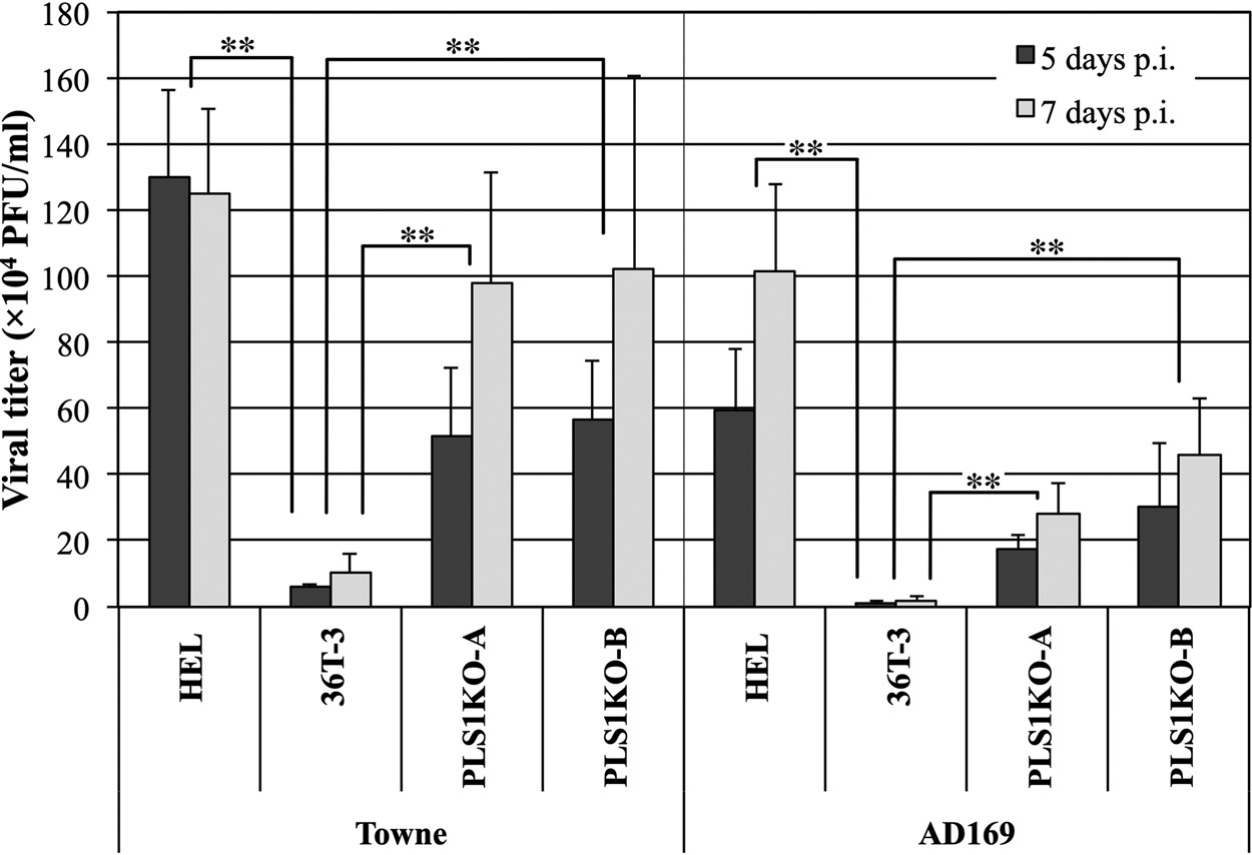
HCMV replicates to higher titers in PLSCR1-KO cells than in parental cells. HEL, 36T-3, PLS1KO-A and PLS1KO-B cells were infected with HCMV at an MOI of 3 PFU per cell. At 5 and 7 days p.i., the supernatants were collected, and viral titers were determined by plaque formation assays in HEL cells from three experiments with duplicate cultures per experiment. Data represent average relative values, and error bars indicate standard deviations. **, *P* < 0.01 by Student's *t* test.

To determine whether PLSCR1 expression affects the levels of HCMV production, the HEL, 36T-3, PLS1KO-A, and PLS1KO-B cell lines were infected with two HCMV strains, Towne and AD169. The culture supernatants were collected at the indicated times after infection, and viral titers were examined using a plaque assay. In Towne infection, although the viral titers in 36T-3 cells were approximately 8% of those in HEL cells even at 7 days p.i., the viral titers produced in both PLS1KO cell lines were approximately 10-fold higher than those in parental 36T-3 cells at 5 and 7 days p.i., and increased to approximately 80% of those in HEL cells at 7 days p.i. (Fig. 4, left). Following AD169 infection, the virus outputs in both infected PLS1KO cell lines were more than 13-fold higher than those in infected 36T-3 cells at 5 and 7 days p.i., although the viral titers produced in both PLS1KO cell lines were less than 50% of those in HEL cells (Fig. 4, right). These results indicated that virus replication was reduced by endogenous PLSCR1 expression. Taken together with the results shown in Fig. 2, these observations suggested that PLSCR1 might impair, in part, the factors and/or functions which influence the quality and quantity of virus production.

### PLSCR1 represses the expression of HCMV major immediate early genes.

**FIG 5:**
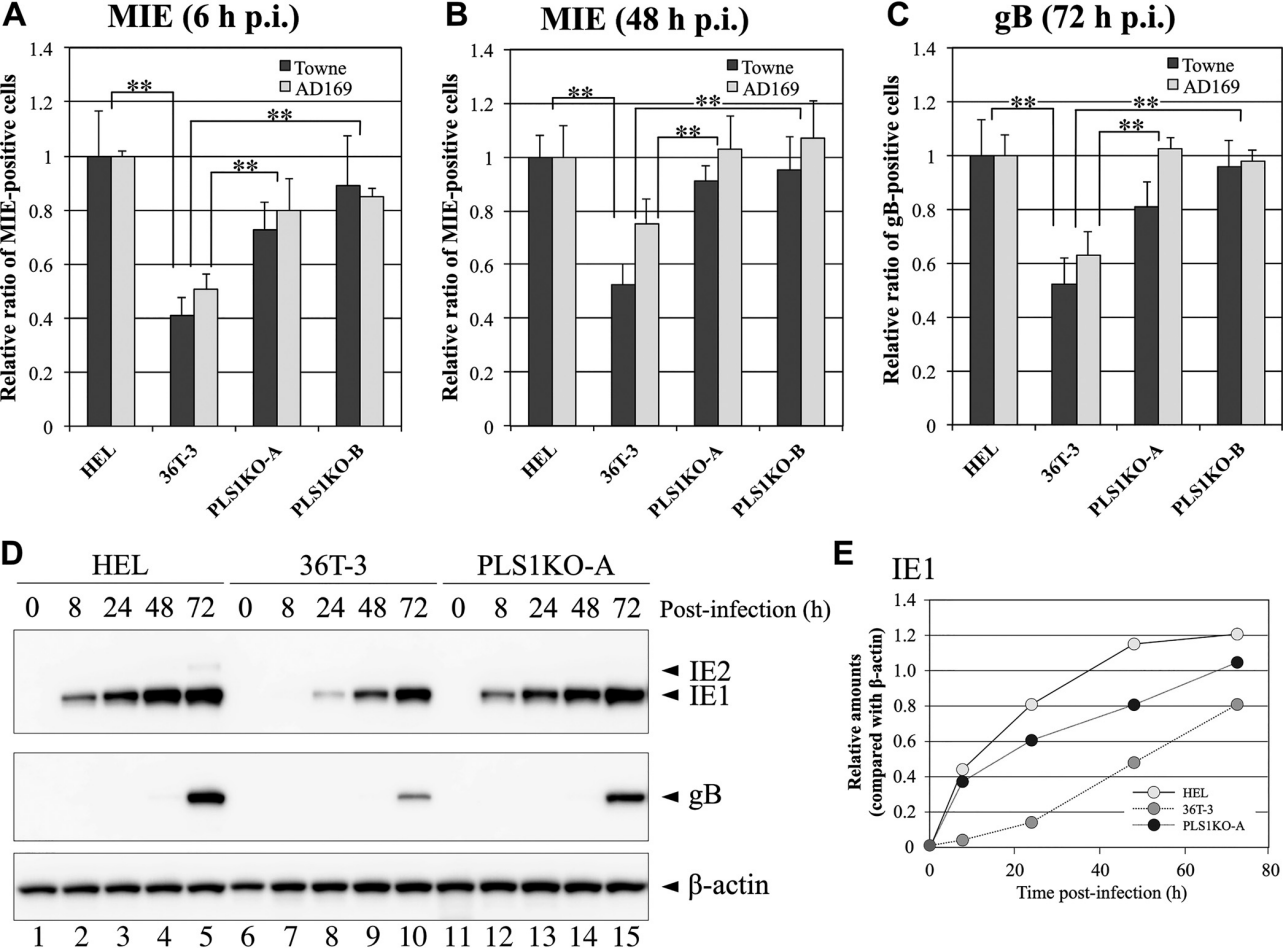
Expression levels of HCMV MIE genes in PLS1KO cells were higher than those in the parental cell line. HEL, 36T-3, PLS1KO-A, and PLS1KO-B cells were infected with HCMV at an MOI of 0.1 PFU per cell. At 6 h (A) or 48 h (B) (for MIE) and 72 h (C) (for gB) p.i., the cells were fixed and then subjected to immunofluorescence staining with an anti-HCMV MIE antibody (A, B) and an anti-HCMV gB antibody (C), respectively. Nuclear and cytoplasmic fluorescence-positive cells were counted as MIE and gB antigen-positive cells, respectively, under a fluorescence microscope. Data represent means and standard deviations of three independent experiments. Data represent average relative values, and error bars indicate standard deviations. **, *P* < 0.01 by Student's *t* test. (D) HEL, 36T-3, and PLS1KO-A cells were infected with the HCMV AD169 strain at an MOI of 3 PFU per cell, and cell lysates were prepared at the indicated time points (hours). Equivalent amounts of lysates were subjected to SDS-PAGE. IB was performed using an anti-HCMV MIE antibody to detect endogenous IE1 and IE2. Detection of endogenous β-actin was performed as described in Fig. 1B and C. (E) Band intensity was quantified as described in Fig. 1B and C.

PLSCR1 has been reported to be involved in virus entry (24–26); however, the results of our virus entry assay demonstrate that PLSCR1 expression does not affect HCMV entry (Fig. S1). HCMV gene expression is temporally regulated, and the major immediate early (MIE) genes are the first and most abundantly expressed, followed by the early genes before DNA replication. To further confirm the PLSCR1-mediated suppression of HCMV growth, the numbers of MIE antigen- and gB antigen (identified as an early gene product)-positive cells in the HEL, 36T-3, PLS1KO-A, and PLS1KO-B cell lines after HCMV infection were determined by immunofluorescence staining. After 6 h of HCMV infection, the numbers of MIE antigen-positive 36T-3 cells were approximately 50% of the numbers of HEL cells (Fig. 5A), and at 48 h p.i., the numbers of MIE antigen-positive 36T-3 cells infected with Towne or AD169 were approximately 50% or 70%, respectively, of the numbers of HEL cells (Fig. 5B). However, the numbers of MIE antigen-positive PLS1KO-A and PLS1KO-B cells were increased to approximately 80% of the number of HEL cells at 6 h p.i. (Fig. 5A) and to levels comparable to those in HEL cells at 48 h p.i. (Fig. 5B). When investigating the gB antigen-positive cells at 72 h p.i., the ratios of the numbers of gB antigen-positive 36T-3, PLS1KO-A, and PLS1KO-B cells versus the number of HEL cells were similar to those obtained with MIE antigen-positive cells (Fig. 5C versus Fig. 5A and B), similar to the plaque formation assay findings (Fig. 2). These observations indicated that endogenous PLSCR1 expression suppresses HCMV growth in 36T-3 cells, probably due to the decrease in MIE expression.

To confirm this PLSCR1-mediated repression of HCMV MIE gene expression, we next evaluated the expression levels of the 72-kDa protein IE1 (UL123), which is one of the proteins encoded by the MIE genes, in HEL, 36T-3, and PLS1KO-A cells after HCMV infection by immunoblot analysis. IE1 was efficiently detectable in HEL and PLS1KO-A cells at 8 h p.i. (Fig. 5D, top panel lanes 2 and 12) but slightly detected in parental 36T-3 cells even 24 h p.i. (Fig. 5D, top panel lane 8), and the protein levels of IE1 in HEL and PLS1KO-A cells were more than 3-fold higher than those in 36T-3 cells at 24 h p.i. (Fig. 5D and E). The 86-kDa protein IE2 (UL122), which is known to be another MIE protein, was slightly detected in only HEL cells at 72 h p.i. (Fig. 5D). In addition, the expression levels of gB were significantly higher in HEL and PLS1KO-A cells than in 36T-3 cells at 72 h p.i. (Fig. 5D, middle panel).

These observations suggest that endogenous PLSCR1 expression suppresses and/or delays the expression of HCMV MIE and early proteins after HCMV infection, resulting in reduced or delayed virus replication.

### PLSCR1 interacts with the HCMV immediate early proteins IE1 and IE2.

**FIG 6:**
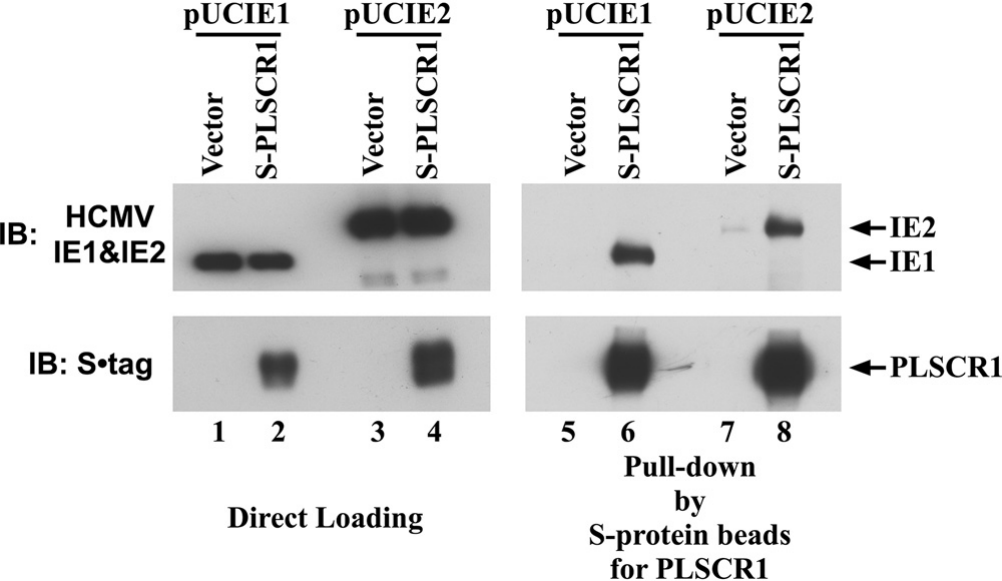
PLSCR1 specifically interacts with HCMV IE1 and IE2. HEK-293 cells (1 × 10^6^) were plated in 60-mm cell culture plates and transfected with 2 μg of pcDNA3 or S-PLSCR1 and 0.5 μg of pUCIE1 or 3 μg of pUCIE2. A total of 300 μg of total cell lysate prepared in NP-40 lysis buffer was incubated with S-protein beads to precipitate PLSCR1. Following pulldown assays, 1 μg (IE1) or 5 μg (IE2) of total cell lysate and the precipitated complexes were divided into two parts and subjected to SDS-PAGE. IB was performed using an anti-S tag antibody to detect PLSCR1 or an anti-HCMV MIE antibody to detect IE1 and IE2.

PLSCR1 has been reported to inhibit the functions of viral transactivators, including human T cell leukemia virus (HTLV)-1 Tax, human immunodeficiency virus (HIV)-1 Tat, human hepatitis B virus (HBV) HBx, and Epstein-Barr virus (EBV) BZLF1 through direct interactions (19, 21–23). It is possible that PLSCR1 also interacts with and negatively regulates the corresponding HCMV-encoded protein, which stimulates viral gene expression. To determine whether PLSCR1 interacts with the IE1 and IE2 proteins, S epitope-tagged full-length human PLSCR1 (S-PLSCR1) was expressed in HEK-293 cells, together with IE1 or IE2. Immunoblot analysis of the total cell lysates indicated that IE1 and IE2 were expressed at similar levels in the presence and absence of PLSCR1 (Fig. 6, lanes 1 to 4). However, immunoblot analysis of the complexes containing PLSCR1 revealed that IE1 and IE2 were efficiently coprecipitated with PLSCR1 (Fig. 6, lanes 5 to 8). This observation revealed that PLSCR1 specifically interacts with HCMV IE1 and IE2 *in vivo*.

### PLSCR1 decreases the levels of the CREB•IE2 and CBP•IE2 complexes.

**FIG 7:**
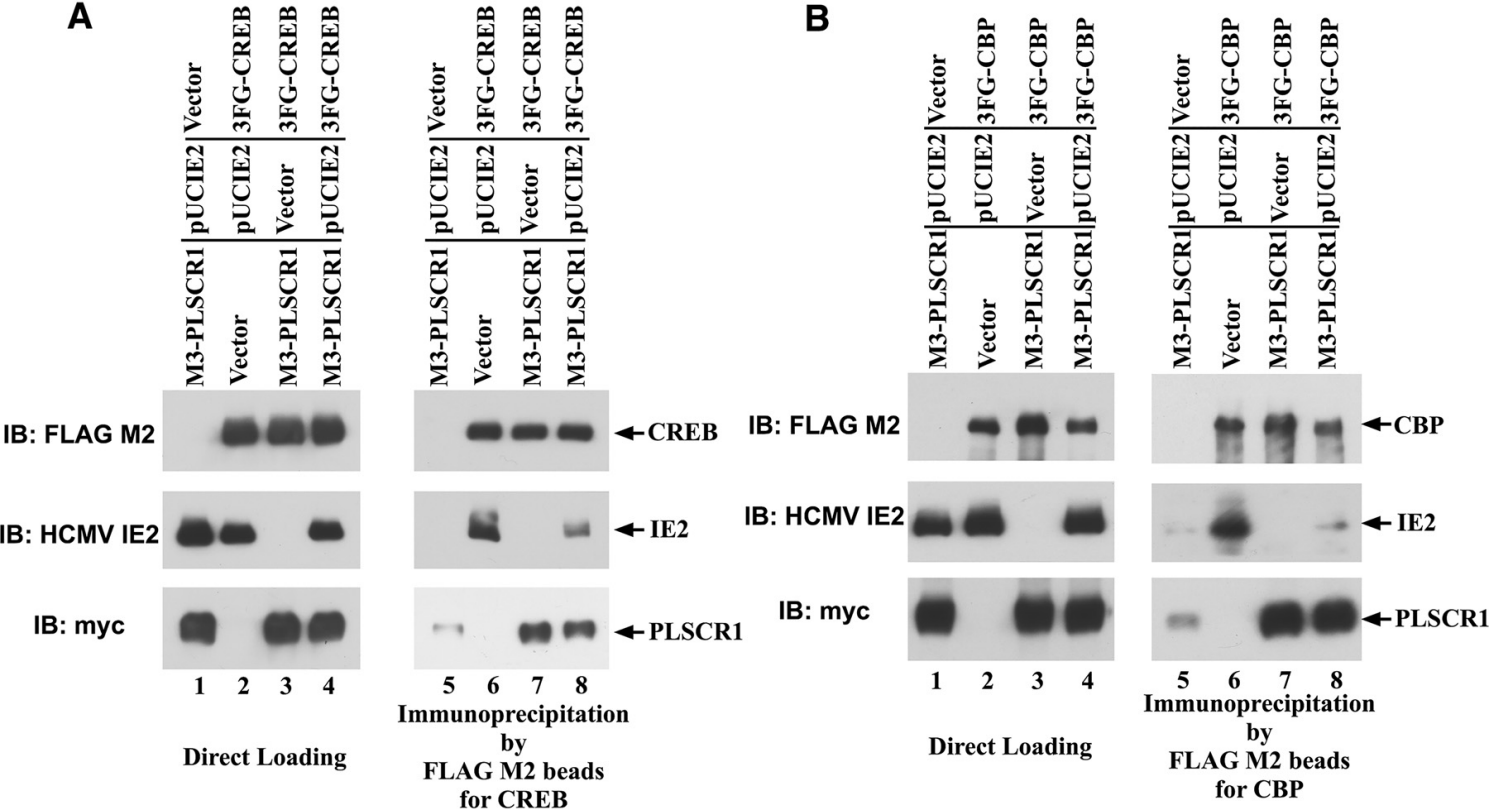
PLSCR1 decreases the levels of the CREB•IE2 and CBP•IE2 complexes. (A) HEK-293 cells (3 × 10^6^) were plated in 90-mm cell culture plates and transfected with 2 μg of pcDNA3 or 3FG-CREB, 1 μg of pcDNA3 or pUCIE2, and 5 μg of pcDNA3 or 3M-PLSCR1. A total of 160 μg of total cell lysate prepared in NP-40 lysis buffer was incubated with FLAG M2 beads to precipitate CREB. Following immunoprecipitation, 15 μg of total cell lysate and the precipitated complexes were divided into three parts and subjected to SDS-PAGE. IB was performed using a FLAG M2 antibody to detect CREB, an anti-myc-tag antibody to detect PLSCR1, or an anti-HCMV MIE antibody to detect IE2. To detect mouse-derived primary antibodies, Mouse TrueBlot ULTRA: Anti-mouse Ig HRP was used as a secondary antibody. (B) HEK-293 cells (4.5 × 10^6^) were plated into 90-mm cell culture plates and transfected with 4 μg of pcDNA3 or 3FG-CBP, 0.75 μg of pcDNA3 or pUCIE2, and 3 μg of pcDNA3 or 3M-PLSCR1. A total of 1,300 μg of total cell lysate prepared in NP-40 lysis buffer was incubated with FLAG M2 beads to precipitate CBP. Following immunoprecipitation, 25 μg of total cell lysate and the precipitated complexes were divided into three parts and subjected to SDS-PAGE. IB was performed as in panel A, except that a FLAG M2 antibody was used to detect CBP.

IE2 is critical for efficient viral replication (27, 28), and the formation of the CREB•IE2 and CBP•IE2 complexes has been suggested to play an important role in the IE2-mediated transactivation of the viral early promoter (29, 30). To determine whether PLSCR1 affects CREB•IE2 complex formation, HEK-293 cells were transfected with 3×FLAG epitope-tagged full-length human CREB (3FG-CREB), three copies of myc epitope-tagged full-length human PLSCR1 (3M-PLSCR1), and IE2. Immunoblot analysis of total cell lysates indicated that the expression levels of CREB were almost identical in the presence and absence of IE2 (Fig. 7A, lanes 2 to 4). However, immunoprecipitation analysis using FLAG M2 beads to precipitate CREB revealed that PLSCR1 expression significantly decreased IE2 coprecipitation with CREB (Fig. 7A, lanes 6 and 8). Furthermore, PLSCR1 was efficiently coprecipitated with CREB in the absence of IE2 (Fig. 7A, lane 7), and IE2 expression also decreased the coprecipitation of PLSCR1 with CREB (Fig. 7A, lane 8).

Next, to determine whether PLSCR1 also affects CBP•IE2 complex formation, HEK-293 cells were transfected with 3×FLAG epitope-tagged full-length human CBP (3FG-CBP), encoding amino acids 2 to 2442 and containing at least two IE2-binding sites (31), 3M-PLSCR1 and IE2. Immunoblot analysis of total cell lysates indicated that the expression levels of CBP were similar to those of IE2 in the presence and absence of PLSCR1 (Fig. 7B, lanes 2 and 4), although the expression of CBP was significantly higher in the absence of IE2 (Fig. 7B, lane 3). However, immunoprecipitation analysis using FLAG M2 beads to precipitate CBP revealed that PLSCR1 expression significantly decreased IE2 coprecipitation with CBP (Fig. 7B, lanes 6 and 8). Furthermore, PLSCR1 was efficiently coprecipitated with CBP in the absence of IE2 (Fig. 7B, lane 7).

These observations indicated that PLSCR1 suppressed the formation of the CREB•IE2 and CBP•IE2 complexes through its interactions with CREB, CBP, and IE2.

### PLSCR1 represses transcription from CRE- and HCMV MIE promoter-regulated reporter constructs.

**FIG 8:**
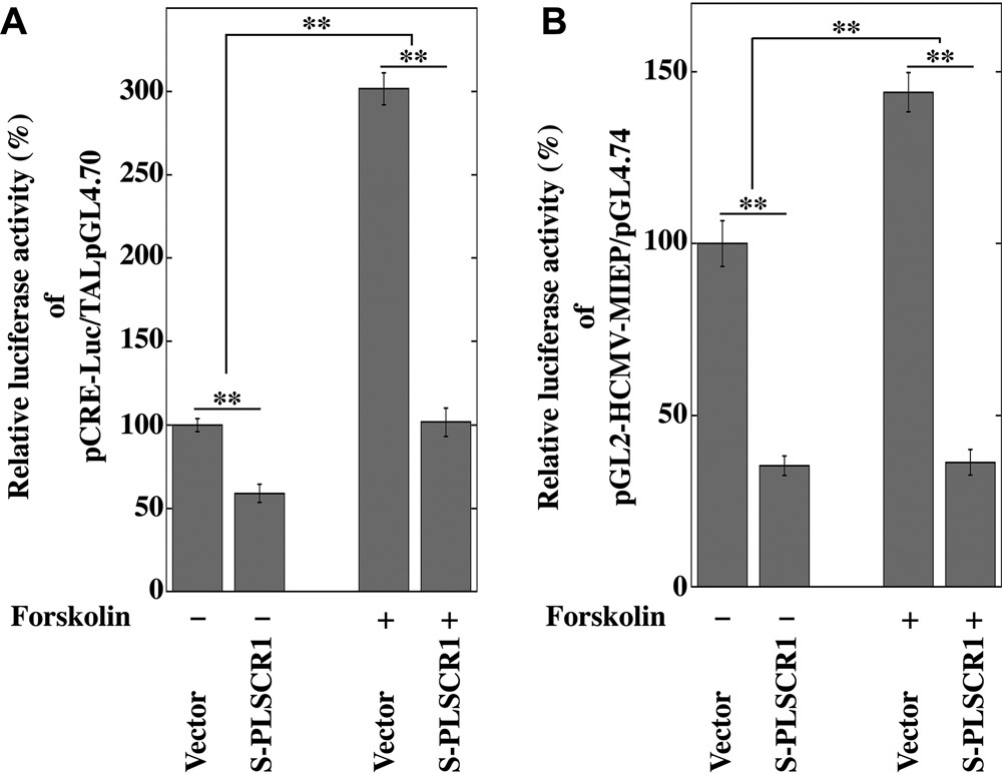
PLSCR1 represses transcription from the CRE- and HCMV MIEP-regulated reporter constructs. (A) HEK-293 cells (1 × 10^5^) were plated in 24-well cell culture plates and transfected with 40 ng of CRE-responsive reporter plasmid (pCRE-Luc), 40 ng of TAL-pGL4.70 (53), and 300 ng of pcDNA3 or S-PLSCR1 using jetPEI. After 24 h of transfection, the cells were treated with or without 5 μM Forskolin (Cayman Chemical) for 16 h. Then the cells were lysed, and their luciferase activity levels were determined. The firefly luciferase/Renilla luciferase activity ratio of cells transfected with the vector only was defined as 100%. Data represent the average relative values from triplicates, and error bars indicate standard deviations. **, *P* < 0.01 by Student's *t* test. (B) HeLa-PLSKO cells (5 × 10^4^) were plated in 24-well cell culture plates and transfected with 2 ng of pMIEP-Luc, 40 ng of pGL4.74 (Promega), and 350 ng of pcDNA3 or S-PLSCR1 using TransIT-X2. After 24 h of transfection, the cells were treated with or without 5 μM Forskolin for 16 h. Then the cells were lysed, and their luciferase activity levels were determined. The firefly luciferase/Renilla luciferase activity ratio of cells transfected with the vector only was defined as 100%. Data represent average relative values from triplicates, and error bars indicate standard deviations. **, *P* < 0.01 by Student's *t* test.

PLSCR1 interacts with CREB in the absence of IE2 (Fig. 7A), and CREB has been shown to play a major role in transcription from HCMV MIE promoter (MIEP) (32, 33). To investigate whether PLSCR1 affects CREB-mediated transcription, we measured the activity of a luciferase reporter plasmid under the control of a synthetic CRE-regulated promoter (pCRE-Luc, Clontech) with Forskolin, which activates CREB-mediated transcription, in the presence of S-PLSCR1 in HEK-293 cells. PLSCR1 overexpression reduced the basal luciferase activity to approximately 60% of that observed in the empty vector-transfected cells (Fig. 8A, lanes 1 and 2). However, the luciferase activity was increased approximately 3-fold by the activation of CREB in the presence of 5 μM Forskolin (Fig. 8A, lanes 1 and 3), and PLSCR1 overexpression efficiently reduced this Forskolin-mediated activation of luciferase activity to approximately 1.5-fold (Fig. 8A, lanes 2 and 4). This observation indicated that PLSCR1 expression repressed CREB-mediated transactivation.

Next, to determine whether PLSCR1 also affects transcription from HCMV MIEP, we measured the activity of a luciferase-reporter plasmid under the control of HCMV MIEP (pMIEP-Luc, [33]) in the presence of S-PLSCR1 in PLSCR1-knockout HeLa cells (HeLa-PLSKO cells, [19]). PLSCR1 overexpression reduced the basal luciferase activity to approximately 35% of that observed in the empty vector-transfected cells (Fig. 8B, lanes 1 and 2). However, the luciferase activity was increased approximately 1.5-fold in the presence of 5 μM Forskolin (Fig. 8B, lanes 1 and 3), and PLSCR1 overexpression efficiently reduced this Forskolin-mediated activation of luciferase activity to near-basal levels (Fig. 8B, lanes 2 and 4). These observations indicated that PLSCR1 expression repressed the CREB-mediated activation of transcription from HCMV MIEP.

Taken together, these observations strongly suggest that PLSCR1 expression represses transcription from MIEP by inhibiting CREB function.

## DISCUSSION

Type I and type II IFNs are known to inhibit HCMV replication (6, 7, 34), and several ISGs have been suggested to be involved in this suppression (9–12). This work revealed for the first time that PLSCR1, whose expression is induced by type I and type II IFNs (18), effectively suppresses HCMV replication using PLSCR1-KO human fibroblast cell lines. PLSCR1 has been reported to enhance the IFN-dependent induction of ISG expression (18). However, our previous observation (19) and this work (Fig. 3) indicated that PLSCR1 is not involved in the IFN-α-mediated induction of ISG15 expression by using PLSCR1-KO cell lines, and it is unlikely that other ISGs are involved in the PLSCR1-mediated suppression of HCMV replication.

This work revealed that PLSCR1 inhibits HCMV replication by repressing MIE protein expression, independent of the virus entry step (Fig. 5 and Fig. S1 in the supplemental material). CRE and CREB are known to play major roles in transcription from HCMV MIEP (33). This work revealed that PLSCR1 forms a complex with CREB and its transcriptional coactivator CBP *in vivo* (Fig. 7) and that PLSCR1 expression represses transcription from CRE- and HCMV MIEP-regulated reporter constructs (Fig. 8). Because PLSCR1 is known to repress the function of the viral transactivator by preventing active complex formation (19, 22), PLSCR1 may repress CREB-mediated transcription by preventing CREB•CBP complex formation. Furthermore, two major IE proteins, IE1 and IE2, play important roles in HCMV lytic replication. IE2 in particular is an essential transactivator of the expression of HCMV early genes (27, 28). However, IE1 is required only for the synergistic transactivation of the early genes with IE2 at a low multiplicity of infection (MOI) (35). This work also revealed that PLSCR1 interacts with IE1 and IE2 *in vivo* (Fig. 6). Because IE1 and IE2 are expressed by alternative splicing from the same mRNA and share 85 amino acids in their N termini, this common region may be involved in the interaction with PLSCR1. Interestingly, the interactions of IE2 with CREB and CBP are known to play an important role in IE2-mediated transactivation of the viral early promoter (29, 30), and PLSCR1 was observed to decrease the levels of the CREB•IE2 and CBP•IE2 complexes (Fig. 7). Overall, interaction-mediated repression of CREB function and/or disruption of the CREB•IE2 and CBP•IE2 complexes may be key for the PLSCR1-mediated suppression of viral replication.

This work also revealed that PLSCR1 forms a complex with CBP in the absence of IE2 (Fig. 7). Interestingly, other PLSCR1-interacting viral proteins, namely, HTLV-1 Tax, HIV-1 Tat, HBV HBx, and EBV BZLF1, also interact with CBP to exert their gene regulatory functions (36–39). CBP is known to interact with a wide variety of cellular proteins through its intrinsically disordered (ID) regions (40), and PLSCR1 also interacts with the target viral proteins through its ID regions (19, 22, 23). Therefore, the ID regions of both proteins may be involved in this interaction, and the interaction of PLSCR1 with CBP through its ID regions may also contribute to the suppression of viral replication and/or transcription by affecting CBP function. Further investigations may elucidate the role of PLSCR1 in viral infection through its interaction with CBP.

The basal expression of PLSCR1 was significantly lower in most human epithelial cell lines, except for epidermal cells, in the absence of IFN treatment (19). This work revealed that the basal expression of PLSCR1 in 36T-3 cells, which are human telomerase reverse transcriptase (hTERT)-immortalized human fibroblasts, was significantly higher than that in HEL cells in the absence of IFN treatment or virus infection (Fig. 1). The precise mechanism of induction of PLSCR1 expression in the absence of IFN signaling remains unclear; however, c-Myc has been suggested to upregulate the expression of PLSCR1 by directly binding to its promoter in HEK-293 cells (41). Because telomerase is reported to mediate c-Myc-dependent gene regulation through direct interaction-mediated stabilization (42) and epidermal cells are known to exhibit high levels of telomerase activity (43), telomerase expression may play a key role in the high expression level of PLSCR1 in 36T-3 cells. In addition, the basal expression of MxA in 36T-3 cells was also higher than that in HEL cells, and HCMV infection decreased the levels of PLSCR1 and MxA at up to 5 h p.i. (Fig. 1). Because the expression of PLSCR1 and MxA is known to be upregulated by IRF-3 (44, 45) and the HCMV tegument protein pp65 (UL83) is suggested to repress IRF-3-mediated gene regulation (46), dysregulation of IRF-3 expression may be involved in the observed HCMV infection-mediated downregulation of PLSCR1 and MxA expression. Interestingly, IRF-3 is reported to be expressed in all epidermal cell layers of human skin (47), which is suggested to express high levels of PLSCR1, and IRF-3 may also be involved in the induction of PLSCR1 expression in 36T-3 cells. Further investigation is warranted to determine whether c-Myc and/or IRF-3 affect PLSCR1 expression in hTERT-immortalizing cells and epidermal cells.

This work revealed that HCMV efficiently replicates in HEL cells with low basal expression levels of PLSCR1. However, in this study, we could not establish PLSCR1-overexpressing HEL cell clones using a plasmid vector because HEL cells have a limited ability to propagate in cell culture and a low transfection efficiency, and we could not determine whether the high level of PLSCR1 expression also represses HCMV replication in HEL cells. Further investigation using transient-transfection with a more efficient gene delivery system may confirm whether the basal expression of PLSCR1 affects HCMV growth not only in 36T-3 cells but also in HEL cells.

## MATERIALS AND METHODS

### Cells and viruses.

Human embryonic lung tissue-derived fibroblasts (HEL) and OUMS-36T-3 (36T-3, an hTERT-immortalized normal human embryo fibroblast cell line, JCRB1006.3) cells were used as HCMV permissive cells. HEL, 36T-3, and HEK-293 cells were maintained in Dulbecco’s modified Eagle’s medium supplemented with penicillin and streptomycin (Nacalai Tesque) and 8% (vol/vol) fetal bovine serum (Sigma-Aldrich) at 37°C in an atmosphere of 5% CO_2_. The laboratory-adapted HCMV strains Towne and AD169 were used.

### Materials.

The antibody MAB810, which recognizes HCMV IE1 and IE2 (48), was purchased from Chemicon. Antibodies against HCMV gB (1-M-12), PLSCR1 (N-17), and actin (I-19) were purchased from Santa Cruz Biotechnology. Antibodies against MxA (N2C2) and ISG15 (GTX121474) were purchased from GeneTex, and Mouse TrueBlot ULTRA: Anti-mouse Ig HRP was purchased from Rockland Immunochemicals. Antibodies against the myc epitope tag (My3) and human IFN-α-2b were purchased from Medical & Biological Laboratories. The anti-S tag antibody and S-protein agarose beads were purchased from Novagen. FLAG M2 antibody and FLAG M2 agarose beads were purchased from Sigma-Aldrich.

### Plasmid construction.

The pUCIE1 and pUCIE2 plasmids expressing the HCMV IE1 and IE2 proteins, respectively, have been previously described (48). S-PLSCR1 and 3M-PLSCR1, which express S epitope-tagged and three copies of myc epitope-tagged full-length PLSCR1, respectively, have also been previously described (22, 49). Human CREB cDNA encoding amino acids 2 to 341 and human CBP cDNA encoding amino acids 2 to 2442 were PCR-amplified from HEK-293 cDNA and cloned into pcDNA3 (Invitrogen), with a 3×FLAG epitope added at the N terminus to produce 3FG-CREB and 3FG-CBP, respectively.

### Transfection.

36T-3, PLS1KO-A, and HeLa-PLSKO cells were transfected with the indicated amounts of DNA using TransIT-X2 (Mirus) as suggested by the manufacturer. HEK-293 cells were transfected with the indicated amounts of DNA using jetPEI (Polyplus Transfection) as suggested by the manufacturer.

### PLSCR1 gene KO.

For Cas9-mediated editing of the PLSCR1 gene, 36T-3 cells (3.5 × 10^5^) were plated in 60-mm cell culture plates and transfected with 3 μg of pCEP-CRISPR-PLSCR1 (19). Next, 48 h after transfection, the cells were selected with 40 μg/mL hygromycin B (Nacalai Tesque) for 2 weeks. Finally, PLSCR1-KO cells were screened as previously described (19).

### Immunoblot analysis.

Immunoblot analysis was carried out using the indicated antibodies as described previously, with modifications (33, 50). Antibody-reactive proteins were detected using ECL Start (Cytiva) and Western blot hyper HRP substrate (Takara Bio) followed by exposure to X-ray film (Fujifilm) and the Fusion Solo 7S Edge chemiluminescence imaging system (Vilber-Lourmat), respectively.

### Plaque assay.

The conditions used for the plaque assay were as described previously, with modifications (51). The cells were grown in 24-well plates to more than 90% confluence and infected with HCMV at MOIs of less than 0.01 PFU/cell. Following 90 min of adsorption, the medium was aspirated from the wells, and fresh medium containing 0.4% agarose was added to duplicate wells. After incubation at 37°C for 6 to 10 days, the cell monolayer was fixed with 10% formalin and then stained with 0.05% methylene blue. Plaques were counted microscopically under low power. The statistical significance was determined using Student's *t* test (Microsoft Excel 2011).

### Indirect immunofluorescence microscopy.

Immunofluorescence microscopy was performed as described previously with modifications (52). Briefly, cells were seeded onto coverslips (13-mm diameter) in a 24-well plate, cultured to more than 90% confluence, and infected with HCMV at an MOI of 0.1 PFU/cell. At the indicated times after infection, the cells were washed twice with phosphate-buffered saline (PBS), fixed for 15 min in a 4% paraformaldehyde-PBS solution, permeabilized for 15 min in a 0.5% Triton X-100 solution, and finally washed twice with PBS. The primary antibodies were diluted in PBS. After incubation with a primary antibody at room temperature for 45 min, the coverslips were washed at least five times and then treated with secondary antibodies. After a further 45-min incubation, the coverslips were washed again at least five times. The cells were mounted using VECTASHIELD mounting medium with 4′,6-diamidino-2-phenylindole (DAPI; Vector Laboratories) and observed by a Zeiss Axio Vert. A1 microscope (Carl Zeiss). Nuclear and cytoplasmic fluorescence-positive cells were counted as MIE and gB antigen-positive cells, respectively, in 10 randomly selected microscopic fields. The statistical significance was determined by using Student's *t* test (Microsoft Excel 2011).

### Pulldown and immunoprecipitation assays using transfected cell lysates.

Forty-eight hours after transfection, cells were harvested and lysed in Nonidet P-40 (NP-40) lysis buffer (19) supplemented with a protease inhibitor cocktail (Nacalai Tesque). Pulldown and immunoprecipitation were performed using S-protein agarose beads (Novagen) and FLAG M2 beads (Sigma-Aldrich) to precipitate the S epitope-tagged and 3×FLAG epitope-tagged proteins, respectively, as previously described (22).

### Dual luciferase assay.

Dual luciferase assays were performed as described previously (53). The statistical significance was determined by using Student's *t* test (KaleidaGraph 4.1.3).
