# Supplementary material for: The Interferon-Inducible Human PLSCR1 Protein Is a Restriction Factor of Human Cytomegalovirus
Source: Microbiol Spectr. 2022 Feb 9;10(1):e01342-21. doi: 10.1128/spectrum.01342-21 (PMC8826943; doi:10.1128/spectrum.01342-21)
Supplement: SUPPLEMENTAL FILE 1 — Supplemental material. Download SPECTRUM01342-21_Supp_1_seq12.pdf, PDF file, 0.2 MB [file spectrum01342-21_supp_1_seq12.pdf]

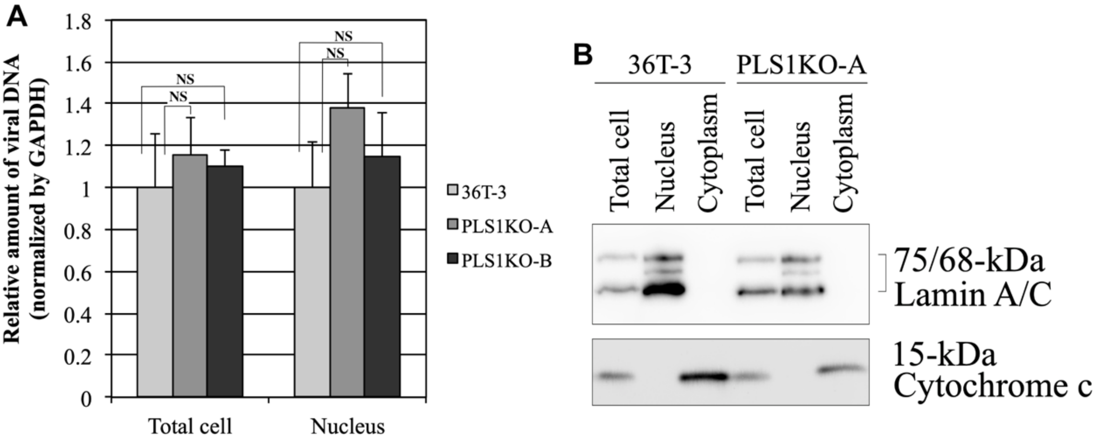

Figure S1. PLSCR1 does not affect HCMV entry. The 36T-3, PLS1KO-A and PLS1KO-B cells (more than 80 % confluency in 6-well plate) were incubated with HCMV AD169 strain at 1 PFU per cell. After 1 h of adsorption period, the residual virus was removed, and the infected cells were further incubated with a maintenance medium. After 4 h of another incubation, cell pellets were collected and divided into four portions. To obtain nuclear pellet, two cell pellets were gently resuspended in 100 µl hypotonic buffer (25 mM Tris-HCl (pH 7.5), 3 mM $MgCl_2$, and 10 mM KCl), and incubated on ice for 3 min. Then, 100 µl of 0.2% NP-40-containing hypotonic buffer was mixed and incubated on ice for 3 min. The cell suspensions were centrifuged for 5 min at 1,500×g at 4°C. The supernatants were saved as the cytoplasmic fractions and the pellets were used as the nuclear pellets. (**A**) The host and penetrated HCMV genomic DNAs were purified from the total cell and nuclear pellets by QIAamp DNA Blood Mini Kit (QIAGEN) according to manufacturer's instructions. Quantitative PCR (qPCR) was performed in triplicate using the CFX96 Touch Real-time PCR Detection system (Bio-rad Laboratories) with GeneAce SYBR qPCR Mix (Nippongene) according to the manufacturer's instructions. One µl of extracted DNAs and a set of primers that were suitable for amplification of human glyceraldehyde 3-phosphate dehydrogenase (GAPDH) (5’-CGAGATCCCTCCAAAATCAA-3’ and 5’-TTCACACCCATGACGAACAT-3’) (1) and HCMV UL54 (5’-GCGCGTACCGTTGAAAGAAAAGCATAA-3’ and 5’-TGGGCACTCGGGTCTTCATCTCTTTAC-3’) (2) were added to a final volume of 10 µl. Relative quantification of the levels of HCMV UL54 DNA were calculated by the $2^{-\Delta\Delta Ct}$ method using CFX Manager Software with host GAPDH DNA as the endogenous reference. Statistical significance was not obtained using Student's t-test (Microsoft Excel 2011). NS, $p>0.05$ by Student’s t-test. The results of qPCR assays indicated that the levels of HCMV DNA were almost identical in the parental and PLSCR1-KO cell lines. (**B**) To obtain total cell and nuclear lysates for SDS-PAGE analysis, cell and nuclear pellets were lysed in SDS-sample buffer (0.25 M Tris-

HCl (pH 6.8), 2% SDS, 10% glycerol, 0.005% bromophenol blue, 5% 2-mercaptoethanol), respectively. Cytoplasmic lysates were prepared from the cytoplasmic fractions by addition of 4-fold concentrated SDS-sample buffer. The same proportion of each lysate was subjected to SDS-PAGE, and immunoblotting analysis was performed with anti-Lamin A/C (E-1, Santa Cruz Biotechnology; as a nuclear marker) and anti-Cytochrome c (A-8, Santa Cruz Biotechnology; as a cytoplasmic marker) antibodies. The data demonstrated the accuracy of nuclear fractionation. These observations indicated that PLSCR1 expression did not affect the entry step of HCMV infection.

References

1. Dussault AA, Pouliot M. 2006. Rapid and simple comparison of messenger RNA levels using real-time PCR. Biol Proced Online 8:1–10.

2. Daikoku T, Saito K, Aihara T, Ikeda M, Takahashi Y, Hosoi H, Nishida T, Takemoto M, Shiraki K. 2013. Rapid detection of human cytomegalovirus UL97 and UL54 mutations for antiviral resistance in clinical specimens. Microbiol Immunol 57:396–399.
